# Neurocognitive function in women affected by the Bhopal gas disaster

**DOI:** 10.4103/0019-5545.46076

**Published:** 2005

**Authors:** R.N. Sahu, G.P. Naik, Asha Dusad, Vikas K. Agrawal

**Affiliations:** * Head and Professor, Department of Psychiatry; *** Trainee in Psychiatry, Department of Psychiatry; **** Trainee in Psychiatry, Department of Psychiatry; ** Professor, Statistics and Demography Department of Community Medicine Hamidia Hospital, Gandhi Medical College, Bhopal, MP

**Keywords:** Methyl isocynate, neurocognitive functions, Bhopal gas disaster

## Abstract

**Background:**

Methyl isocynate (MIC) is a reactive, toxic, volatile and inflammable gas. Exposure to MIC causes neurotoxicity and somatic abnormalities in human beings.

**Aim:**

We compared neurocognitive function in MIC-exposed women and a control group, as well as cognitive function in the MIC group and examined them with reference to age.

**Methods:**

The study sample comprised 30 women and a control group of 30 women. Both the groups were subjected to a detailed neuropsychiatric examination along with assessment of neurocognitive function using the PGI-Battery of Brain Dysfunction (PGI-BBD).

**Results:**

Mean scores of immediate recall, visual retention, difference in performance quotient/verbal quotient, Nahar–Bensen and Bender–Gestalt test were significantly affected in MIC-exposed women. However, among MIC-exposed women, neurocognitive functions were similarly affected in women in various age groups.

**Conclusion:**

Women in the MIC-exposed group had significant neurocognitive dysfunction in some specific areas as compared to women in the control group. The mean score of dysfunction rating of the PGI-BBD showed significant differences in neurocognitive functions between MIC-exposed and non-exposed women.

## INTRODUCTION

On the night of 2 December 1984, 40 tonnes of methyl isocynate (MIC) gas leaked from tank 610 of the Union Carbide India Limited factory in Bhopal into the surrounding environment. This leak of MIC gas, an extremely hazardous chemical, which occurred over a short span of a few hours, covered the city of Bhopal in a cloud of poisonous gas. The estimated mortality from this accident is believed to have been between 2500 and 5000, with up to 200,000 injured.^1^

Although the devastating psychiatric trauma caused by the disaster is well known, serious attention has not been given to it, in spite of significant psychiatric morbidity being reported, e.g. neuroses, anxiety and exacerbation of pre-existing adjustment reaction.^2^

Cytogenetic studies carried out by Ghosh *et al*. after the accident on a sample of 40 exposed males and 40 exposed females revealed a statistically higher frequency of chromosomal aberrations among those exposed compared with non-exposed controls.^3^ These chromosomal aberrations were more pronounced in females.

The present study was carried out to assess the effects of MIC gas on neurocognitive functions among exposed and unexposed women.

## METHODS

This prospective study was carried out from May 2004 to April 2005.

### Study sample

The sample for the present study comprised 30 women, selected randomly from patients attending Kamla Nehru Hospital (a specialized centre for the treatment of gas victims), Jaiprakash Nagar and Quazi Camp. These areas were severely exposed to MIC.

The control group comprised 30 women drawn from the medical college hospital. They were relatives of patients attending/admitted in the medical or surgical wards (excluding psychiatric wards). Care had been taken to ensure that none of the subjects lived within 100 km of the factory, and had never resided in MIC-exposed areas. The study subjects were from Bhopal or nearby areas such as Vidhisha, Sehore, Bairagarh, etc. Both the groups belonged to a low socio-economic status and were matched for age (20–60 years) and education level (at least class 5). The study group was further divided into two age groups—20–40 years and 40–60 years.

Patients who had other organic causes of neurocognitive impairment, e.g. space-occupying lesions, encephalopathy, history of trauma, drug-induced impairment, infections or neurological disorders such as stroke, epilepsy, meningitis and encephalitis were excluded from the study.

### Methodology

A written consent was taken from subjects of both the study and the control groups. Both the groups underwent a detailed neuropsychiatric examination along with assessment of neurocognitive functions. The PGI-Battery of Brain Dysfunction (PGI-BBD) developed by Prasad D and Verma^4^ was administered individually to all subjects. The score obtained was statistically analysed with the appropriate tools to assess the effects of MIC gas on neurocognitive functions.

The components of the PGI-BBD are as follows:

1. PGI Memory Scale (PGIMS)
2. Revised Bhatia Short Battery of Performance Tests of Intelligence (BSR-R)
  Subtests
  —Koh block design
  —Pass a long test
3. Verbal Adult Intelligence Scale (VAIS)
  Subtests
  —Information
  —Digit span
  —Arithmetic
  —Comprehension
4. Nahar–Benson test
5. Bender visual motor Gestalt test (Bender–Gestalt test)

### Statistical analysis

The Student *t* test was used to analyse the difference between mean scores of the PGI-BBD among cases and controls. A p value of <0.05 was considered significant.

## RESULTS

The mean scores of memory dysfunction among the MIC-affected group were higher in all subtests, except attention and concentration, which were higher in the non-MIC group (Table 1). The mean score of immediate recall was 1.25 in the MIC group while it was only 0.4 among the non-MIC group. Similarly, the mean score of visual retention was 1.1 in the MIC group, which was higher in comparison with that of the non-MIC group (0.2). Table 2 gives the results of performance tests of intelligence among the two groups.

**Table 1:** Memory dysfunction in the victims and controls

Subtest of memory	MIC group (mean score)	Non-MIC group (mean score)	*t* value
Remote memory	1.1	0.3	1.66
Recent memory	1.1	0.65	1.21
Mental balance	2.05	1.7	0.88
Attention/concentration	1.2	1.65	1.13
Delayed recall	0.9	0.6	0.87
Immediate recall	1.25	0.4	2.43*S
Retention of similar pair	0.7	0.2	1.73
Retention of dissimilar pair	1	0.9	0.28
Visual retention	1.1	0.2	2.6*S
Recognition	1.6	1.4	0.60

* p<0.05; S: significant

**Table 2:** Dysfunction of performance tests of intelligence

Subtest of intelligence	MIC group (mean score)	Non-MIC group (mean score)	*t* value
P/K × 100	0.8	0.85	0.14
Performance quotient	1.25	1.35	0.28

P: raw score on pass a long test; K: raw score on Koh block design

Table 3 shows the mean scores of total quotient (TQ) on information, digit span, arithmetic and comprehension in both the groups. The mean score in performance quotient/verbal quotient (PQ/VQ) was slightly lower among controls than that of the MIC-affected group.

**Table 3:** Dysfunction of the Verbal Adult Intelligence Scale (VAIS)

Subtest of VAIS	MIC group (mean score)	Non-MIC group (mean score)	*t* value
TQ on information	0.95	0.85	0.29
TQ on digit span	0.8	1.05	0.70
TQ on arithmetic	1.35	1.55	0.49
TQ on comprehension	0.8	0.7	0.19
Difference in PQ/VQ	2.0	1.45	2.30*S

* p<0.05; S: significant

TQ: total quotient; PQ: performance quotient; VQ: verbal quotient

Table 4 gives the results of the Nahar–Benson and Bender–Gestalt tests. The mean score of the dysfunction rating scores in all subtests of the PGI-BBD is given in Table 5.

**Table 4:** Dysfunction of the Nahar–Benson and Bender–Gestalt tests

Subtest of	MIC group (mean score)	Non-MIC group (mean score)	*t* value
Nahar–Benson test	1.25	0.5	2.23* S
Bender–Gestalt test	2.5	1.65	2.95** S

* p<0.05;

** p< 0.01; S: significant

**Table 5:** Mean of the dysfunction rating scores in all subtests of the PGI-BBD in the age group of 20–60 years among the MIC-exposed group (*n*=15)

	Age group (in years)		
		
	20–40	40–60	*t* value
Mean score	22.7	25.0	0.66

Table 6 compares the mean of the dysfunction rating scores in all subtests of the PGI-BBD among the MIC-exposed and non-exposed subjects.

**Table 6:** Mean of dysfunction rating scores in all subtests of the PGI-BBD in the MIC-exposed and non-MIC exposed group (*n*=30)

	Categories		
		
	MIC group	Non-MIC group	*t* value
Mean score	23.85	17.75	2.16* S

* p<0.05; S: significant

## DISCUSSION

Gas victims show a higher level of dysfunction than the normal group in some components of subtests of memory. In remote memory, recent memory, delayed recall, retention of similar pair and visual retention, the level of dysfunction was higher in MIC-exposed women than women in the non-exposed group. Immediate recall and visual retention were significantly more affected (p<0.05) among MIC-exposed women. Mishra *et al*. conducted a clinical psychometric study on MIC-exposed victims that included the Benton visual retention test, Wechsler memory scale and standard progressive matrices (SPMs).^5^ They found that severely affected victims had significantly impaired SPM, associated learning problems, motor speed and neurocognitive function. Gupta *et al*. studied 350 exposed subjects, 2.5 months after the accident occurred, and reported that auditory and visual memory, attention response speed and vigilance were significantly impaired in the MIC- exposed group, in comparison with controls.^6^ No significant difference in intelligence was found.

We found that the dysfunction score on VAIS showed that TQ on information, digit span, arithmetic and comprehension were slightly higher among MIC-affected women but it was not statistically significant. However, the difference in PQ/VQ was significantly higher (p<0.05) among MIC-exposed victims.

One of the most important segments of the PGI-BBD are the Nahar–Benson and Bender–Gestalt tests, in which the perception and reproduction of Gestalt figures are determined by the biological principle of sensory–motor action. The capacity to reproduce Gestalt figures was badly affected in MIC-exposed victims, which was statistically significant (p<0.01) in our study.

The relevance of age with regard to neurocognitive impairment was examined in two age groups—20–40 years and 40–60 years—among MIC-exposed women; no significant difference was noticed in both the age groups. Hence, age does not seem to be a factor affecting neurocognitive functions of MIC-exposed women.

The number of women suffering from neurocognitive dysfunction with organic brain pathology was very high in comparison to the control group in this study. A study on organic brain pathology among MIC-exposed patients found that 52% of patients had signs of organic brain damage in comparison with controls.^7^

In the present study, the dysfunction rating scores of all subtests of the PGI-BBD showed a significant difference (p<0.05) in neurocognitive functions between the MIC-exposed and non-exposed groups. Similarly, Singer and Scott found severe deficits in memory, manual dexterity, mental flexibility and word fluency in all patients after 16 months of exposure to toluene diisocyanate.^8^

## CONCLUSION

The present study concludes that MIC can cause long-term neurocognitive impairment in an exposed population.

Thus, improvement in psychiatric services and psychological rehabilitation are essential for those affected by exposure to MIC. Community-based mental health programmes and group therapy are required to improve the personality and neurocognitive abilities of those living in gas-affected areas.
